# Prognostic value of delayed enhancement cardiovascular magnetic resonance in patients with sarcoidosis

**DOI:** 10.1186/1532-429X-14-S1-O13

**Published:** 2012-02-01

**Authors:** Mahi Ashwath, Han W Kim, Michele Parker, Raymond J Kim

**Affiliations:** 1 Duke University, Durham, NC, USA

## Background

Although sudden death is a leading cause of mortality in patients with sarcoidosis, cardiac involvement is often unrecognized clinically. Delayed enhancement cardiovascular magnetic resonance (DE-CMR) has been shown to detect cardiac involvement in patients with sarcoidosis. We sought to evaluate the prognostic value of myocardial sarcoid involvement by DE-CMR.

## Methods

Consecutive patients with biopsy proven extracardiac sarcoidosis or suspected isolated cardiac sarcoidosis were prospectively recruited. Patients with history of cardiac surgery, heart transplant, documented coronary artery disease, congenital heart disease were excluded. Of the 251 meeting criteria, 231 participants were enrolled (18 declined consent, 2 were minors) underwent CMR, and followed for 2.7±2.2 years for major adverse cardiac events (cardiac death, appropriate defibrillator shock, pacemaker placement, or heart transplantation). CMR images were analyzed by a consensus of two experienced readers who where blinded to all other data.

## Results

**Figure 1:**
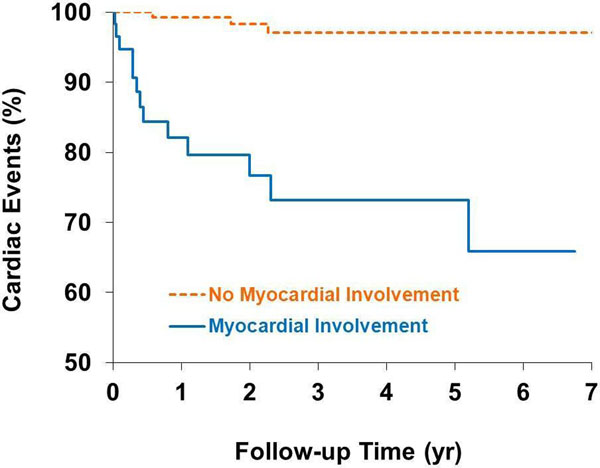


Patients were predominantly middle-aged (48±11 years), female (63%), and African-American (72%). More than half (53%) were on corticosteroid therapy at the time of CMR. Lung (87%) and lymph nodes (45%) were the most common organs of sarcoid involvement. On chest radiography, most were pulmonary stage I /II (66%). Overall, left ventricular ejection fraction (EF) was preserved (58 ±13%). Right ventricular EF was also preserved (49% ±11%). Myocardial involvement (hyperenhancement) on DE-CMR was found in 60 patients (26%). Most (80%, 48/60) had involvement of the interventricular septum. On follow-up, 16 patients had 19 cardiac adverse events, including 6 cardiac deaths, 2 heart transplants, 7 appropriate shocks, and 4 pacemaker implantations for high grade AV block. Patients with myocardial involvement by DE-CMR had a significantly higher rate of adverse cardiac events than patients without (Figure 1). On multivariable Cox analysis including baseline clinical variables and left ventricular EF, the presence of myocardial involvement was the only independent predictor of cardiac events (HR 14.5; 95% CI 4.1, 50.8).

## Conclusions

In patients with sarcoidosis, myocardial involvement by DE-CMR is the strongest independent predictor of adverse cardiac prognosis.

## Funding

None.

